# Adult Respiratory Syncytial Virus Infection: Defining Incidence, Risk Factors for Hospitalization, and Poor Outcomes, a Regional Cohort Study, 2016–2022

**DOI:** 10.3390/pathogens13090750

**Published:** 2024-08-31

**Authors:** Tal Brosh-Nissimov, Daniel Ostrovsky, Amos Cahan, Nir Maaravi, Daniel Leshin-Carmel, Nitzan Burrack, Rotem Gorfinkel, Lior Nesher

**Affiliations:** 1 Faculty of Health Sciences, Ben Gurion University of the Negev, Beer Sheba 84101, Israel; ostrdani@post.bgu.ac.il (D.O.); amosc@assuta.co.il (A.C.); nirma@assuta.co.il (N.M.); danielles@assuta.co.il (D.L.-C.); rotemgur@clalit.org.il (R.G.); 2 Infectious Diseases Unit, Samson Assuta Ashdod University Hospital, Ashdod 7747629, Israel; 3 Clinical Research Center, Soroka University Medical Center, Ben-Gurion University of the Negev, Beer Sheba 84101, Israel; 4 Infectious Disease Institute, Soroka University Medical Center, Beer Sheba 84101, Israel

**Keywords:** respiratory syncytial viruses, incidence, hospitalization, adult, epidemiology, influenza, human

## Abstract

Background: Respiratory syncytial virus (RSV) is a significant cause of illness in adults, especially older adults and those with underlying conditions. This study aimed to assess the incidence of RSV hospitalizations in adults and identify risk factors for hospitalization and poor outcomes. Methods: A retrospective cohort study was conducted using data from two hospitals in southern Israel from 2016–2022. We calculated incidence rates of RSV and influenza hospitalizations. Risk factors for hospitalization were analyzed using Poisson regression. We evaluated poor outcomes (death, ICU admission, or mechanical ventilation) among RSV-hospitalized patients. Results: The median annual incidence of RSV hospitalization was 28.2/100,000 population, increasing with age to 199/100,000 in those ≥75 years. Significant risk factors for RSV hospitalization included pulmonary diseases (RR 4.2, 95% CI 3.4–5.2), cardiovascular diseases (RR 3.3, 95% CI 2.6–4.2), and chronic renal failure (RR 2.9, 95% CI 2.3–3.7). Among hospitalized RSV patients, 13.9% had poor outcomes. Renal failure (RR 1.81, 95% CI 1.23–2.66), neutropenia (RR 2.53, 95% CI 1.19–5.35), neutrophilia (RR 1.66, 95% CI 1.81–2.34), and lymphopenia (RR 2.03, 95% CI 1.37–3.0) were associated with poor outcomes. Conclusions: RSV causes a substantial burden of hospitalizations in adults, particularly among older adults and those with comorbidities. Identifying high-risk groups can help target prevention and treatment strategies, including vaccination.

## 1. Introduction

Respiratory syncytial virus (RSV) is a significant cause of respiratory illness in adults, especially among older adults and those with underlying health conditions. While RSV is often associated with children, its impact on adults, particularly in terms of hospitalizations and severe outcomes, has received increasing attention in recent years.

In the United States, it is estimated that RSV causes between 60,000 and 160,000 hospitalizations annually among adults aged 65 years and older, leading to substantial morbidity and mortality [1]. The incidence of RSV hospitalizations varies significantly with age and underlying health conditions. For example, Zheng et al. reported that the estimated annual incidence of respiratory hospitalizations due to RSV was highest among older adults, particularly those aged 75 years and above [2]. Another study highlighted that the incidence rates of RSV hospitalizations among adults aged 60 years and older ranged from 236 to 363 per 100,000 person-years for respiratory hospitalizations and from 584 to 912 per 100,000 person-years for cardiorespiratory hospitalizations [3]. Among adult patients hospitalized with viral respiratory infections, RSV was less common than influenza (FLU) and coronavirus infection disease 19 (COVID-19) but had worse outcomes [4].

Several risk factors have been suggested to increase the likelihood of severe outcomes in adults hospitalized with RSV [5]. Age is a primary risk factor, with older adults, particularly those aged 75 years and above, being at a higher risk of severe disease. Pre-existing comorbid conditions also play a significant role. Chronic heart and lung diseases, such as chronic obstructive pulmonary disease (COPD) and congestive heart failure (CHF), are common among adults hospitalized with RSV and are associated with worse outcomes. Immunocompromised individuals, including those with conditions such as diabetes and kidney disease and those undergoing immunosuppressive treatments, are also at increased risk. Understanding the incidence rates and risk factors for poor outcomes in adults hospitalized with RSV is crucial for developing targeted prevention and treatment strategies.

In recent years, significant progress has been made in developing vaccines for RSV in adults. In 2023, the U.S Food and Drug Administration (FDA) approved two vaccines: the adjuvanted RSVPreF3 vaccine (Arexvy, GSK) and the RSVpreF vaccine (Abrysvo, Pfizer) for people aged 60 and older. Since these vaccines are costly, identifying the individuals who would benefit the most will help healthcare providers determine and prioritize the population that should receive the vaccine.

Within our distinctive clinical environment, we endeavor to assess and determine the prevalence of RSV illnesses in the adult demographic that result in hospital admissions. Furthermore, our objective is to identify high-risk groups that would benefit from targeted vaccination efforts. 

## 2. Methods

### 2.1. Study Setting and Design 

This study was a retrospective cohort analysis based on data from two healthcare institutions in the southern region of Israel: Soroka University Medical Center (SUMC) and Assuta Ashdod University Hospital (AAUH) from 1 September 2016, to 31 August 2022. Both are the sole inpatient healthcare institutions in their area. Therefore, we assumed that the data on hospitalized patients represent all admissions for the population. Both institutions perform routine influenza (FLU) and RSV screening using commercial PCR multiplex assays such as Xpert^®^ Xpress Flu/RSV by Cepheid (Sunnyvale, CA, USA) and Allplex^®^ RV Essential Assay by Seegene (Seoul, Republic of Korea) on all patients with any signs of acute respiratory disease during the fall and winter seasons upon admission.

The analytical framework encompassed two distinct strata:

**Population-level evaluation**: This stratum quantifies the hospitalization incidence due to FLU and RSV and identifies associated risk determinants.

**Institutional-level evaluation**: This stratum focuses on elucidating risk determinants for poor outcomes among hospitalized individuals with RSV.

### 2.2. Data Sources 

We calculated the incidence rate of FLU- and RSV-positive hospitalizations for the population-level stratum. The numerator comprised aggregated data of all FLU- and RSV-positive hospitalizations from SUMC and AAUH electronic health records (EHR). The denominator was the population size of both areas per age strata, derived from the Israel Central Bureau of Statistics. Data for Analysis of risk factors for hospitalization were extracted from the Clalit Data sharing platform powered by MDClone^®^ Beer Sheba, Israel, a subsidiary of Clalit Health Services (CHS), the largest health maintenance organization in Israel. The MDclone system aggregates inpatient and outpatient EHR data, ensuring precise comorbidity characterizations. This analysis was restricted to CHS patients hospitalized in SUMC. For the institutional-level stratum, data, including demographic, clinical, and laboratory parameters, were extracted from the EHRs of both institutions.

### 2.3. Patients and Definitions

We included all hospitalized patients ≥ 18 years of age who had a positive polymerase chain reaction (PCR) for FLU or RSV within three days of admission. Excluded were FLU/RSV coinfections and repeat PCR-positive patients within 30 days. 

Risk factor delineation adhered to the criteria set forth by the Elixhauser comorbidity index, based on the International Classification of Diseases, Ninth Revision (ICD-9) codification (Supplementary Table S1). We extracted vital signs and laboratory indices within a 24-h window surrounding the index PCR result. In instances of multiple data points, the protocol mandated the extraction of the highest recorded values for fever and neutrophil count and the lowest oxygen saturation in ambient air and lymphocyte counts. We created a composite endpoint of a poor outcome for RSV, including inpatient mortality, intensive care unit (ICU) admission, or the requirement for mechanical ventilation.

### 2.4. Statistical Methods

Continuous variables were described by the means ± standard deviation (SD), median, minimum, and maximum and compared between the study groups using a *t*-test or a Wilcoxon test, depending on their distribution. Categorical variables were presented by the percentage of available cases and compared between study groups using a chi-square or Fisher’s exact test. For the calculation of the annual RSV incidence, we used medians in order to minimize the impact of low RSV circulation during the early COVID-19 pandemic (2020–2021). Data were extracted via the MDclone system to assess the association between comorbidities and hospitalizations attributable to RSV or FLU. This process was iteratively conducted six times, with each iteration anchored to the first of September of the respective year, enabling annual updates of comorbidity status preceding the winter season. A multiple quasi-Poisson regression model was employed to compute the relative risk of hospitalization, with adjustments made for age demographics. The model accounted for repeated measures by incorporating a random intercept corresponding to each patient’s identification number.

A multivariable quasi-Poisson regression was performed to evaluate factors associated with poor inpatient outcomes. We conducted the regression twice to assess the neutrophil and lymphocyte count on a continuous scale and as a categorical variable using acceptable cutoffs. To allow for non-linearity and ensure interpretability, we used a natural cubic spline, and degrees of freedom were determined based on Bayesian information criteria. The adjusted predictions were computed from the model by setting the non-focal variables on their mean. The significance threshold was set to 5% and aligned with a 95% confidence interval (CI) presented throughout the analysis. All analyses were conducted using R (R version 4.3.3, The R Project for Statistical Computing r-project.org).

This study was reviewed and approved by the ethics committees of SUMC and AAUH (SOR-23-39 and AA-23-0013), and both committees granted a waiver of informed consent. We followed the Strengthening the Reporting of Observational Studies in Epidemiology (STROBE) guidelines [6]. 

## 3. Results

**Figure 1:**
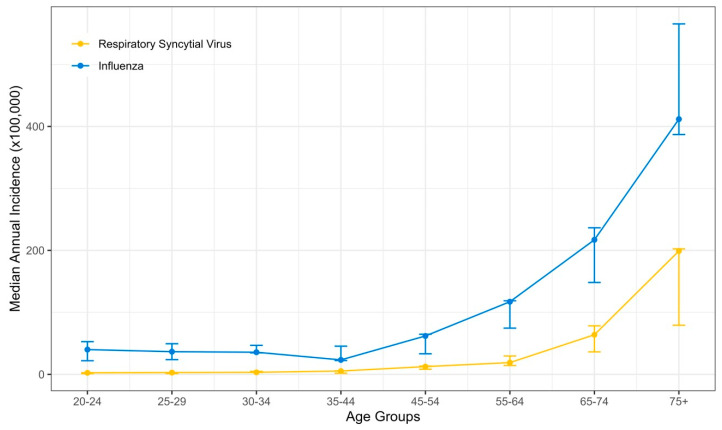
The median annual incidence of RSV- and FLU-positive hospitalizations by age groups between 2016–2022. Error bars represent the interquartile range.

During the study period, 3941 and 989 hospitalized adult patients tested positive for influenza and RSV, respectively; we excluded 29 co-infected patients. The median annual incidence rate for influenza hospitalization was 101.6/100,000 population (interquartile range [IQR] 79.3–114.3), whereas for RSV, the median incidence was 28.2/100,000 population (IQR 15.6–29.5). The median incidence rates for hospitalizations with poor outcomes were 12.1/100,000 population (IQR 8.9–12.5) for FLU and 2.8/100,000 population (IQR 2.4–3.4) for RSV. An increase in hospitalizations and those with poor outcomes was observed with increasing age, commencing at 45 years (Figure 1 and Table S2). RSV hospitalization incidence rates were 63.9 (IQR 36.3–78.3) for ages 65–74 and highest for patients 75 years and older, reaching 199 (IQR 79.2–202.4).

### 3.1. Risk Factors Associated with FLU- and RSV-Positive Hospitalizations 

The demographic and health characteristics of the CHS-insured population and those hospitalized with FLU or RSV are presented in Supplementary Table S3. The population size ranged between 353,398 and 403,686 during the study period, and annual hospitalization numbers ranged between 0–533 for FLU and 18–118 for RSV. Supplementary Table S4 provides a comparative analysis of demographic and clinical characteristics between FLU- and RSV-hospitalized patients. The main differences included an older age, with a median of 73.6 years (IQR 62.6–81.8) for RSV vs. 68.4 (IQR 52.6–79.4) for FLU (*p* < 0.001); a smaller proportion of males, with 39.8% for RSV vs. 47.3% for influenza (*p* = 0.003); a higher percentage of Jewish individuals, with 74.8% for RSV vs. 67.3% for FLU, and a higher rate of comorbidities, with 91.3% of RSV patients having at least one comorbidity, vs. 83.0% for FLU (*p* < 0.001). 

**Figure 2:**
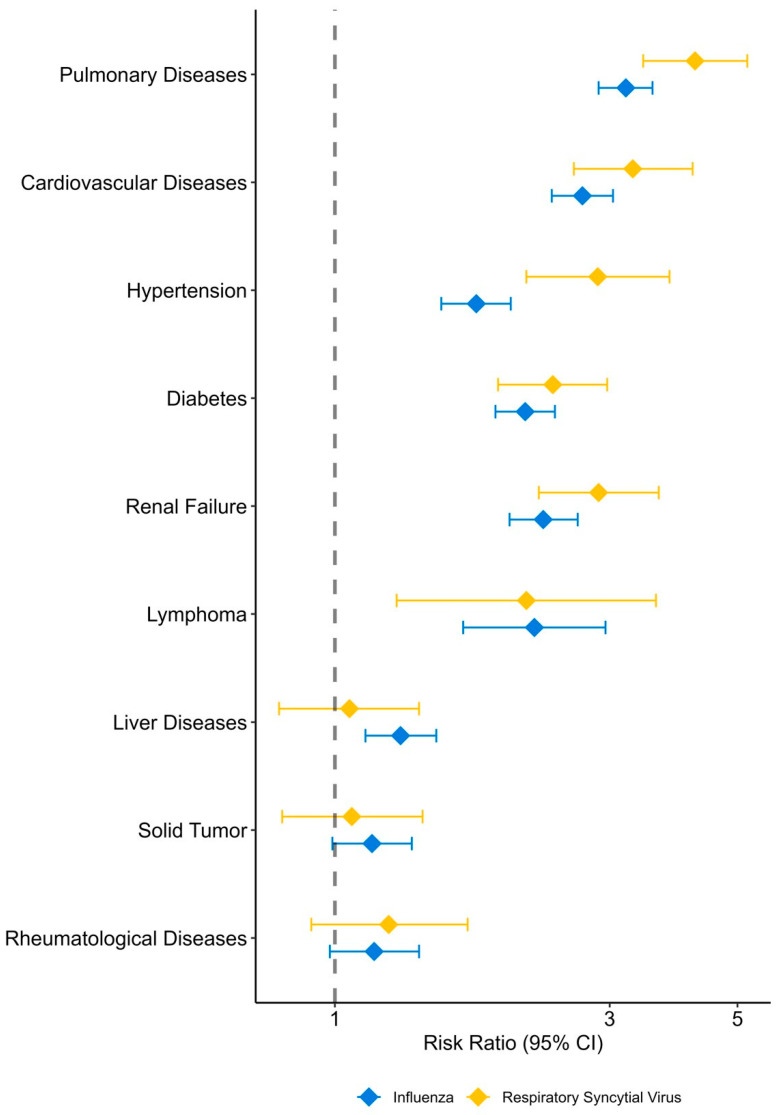
Risk factors associated with hospitalization due to influenza and respiratory syncytial virus, adjusted for age.

Figure 2 presents the age-adjusted hospitalization risk factors due to FLU or RSV. The estimates are provided in Supplementary Table S5. Most analyzed comorbidities were associated with a significantly higher risk of both FLU and RSV hospitalizations, with the following risk ratios (RRs) in a decreasing order: pulmonary diseases, cardiovascular diseases, hypertension, diabetes, renal failure, lymphoma, and rheumatoid diseases. For solid tumors and liver diseases, there was a modestly elevated risk of FLU hospitalization and a non-significantly increased risk of RSV hospitalization.

### 3.2. Risk Factors Associated with Poor Outcomes of an RSV Hospitalization

Among hospitalized patients, 13.9% (n = 131) with RSV developed a composite poor outcome. Table 1 presents the characteristics of patients hospitalized with RSV with good and poor outcomes. Patients with poor outcomes had more diabetes mellitus (DM) and chronic renal failure (CRF) (44.3% vs. 33.2%, *p* = 0.017 and 27.5% vs. 15%, *p* = 0.001, respectively), and there was a marginally significant higher proportion of elderlies (77.9% vs. 70.2%, *p* = 0.066). There were no significant differences between patients with good and poor outcomes in reference to median age, sex, other comorbidities, or having fever. 

Patients with poor outcomes presented with higher white blood cell (WBC) counts, higher neutrophil counts, and lower lymphocyte counts. Hypoxemia at presentation (defined as an oxygen saturation at room air of ≤93%) was also marginally more common in patients with a poor prognosis (45.5% vs. 35.5%, *p* = 0.089).

More patients with a poor outcome received antimicrobial treatment than those with a good outcome: 57.5% vs. 42.8% (*p* = 0.003) before the results of RSV-PCR and 53.5% vs. 23.8% (*p* < 0.001) after the results. 

The composite poor outcome was mainly composed of in-hospital mortality (59 of 131 patients), with fewer patients transferred to the ICU (37/131) and/or mechanically ventilated (36/131). Rehospitalization within one month was uncommon for patients with a good outcome (8.9%), whereas 17/72 (23.6%) of those with a poor outcome that survived were re-admitted.

**Figure 3:**
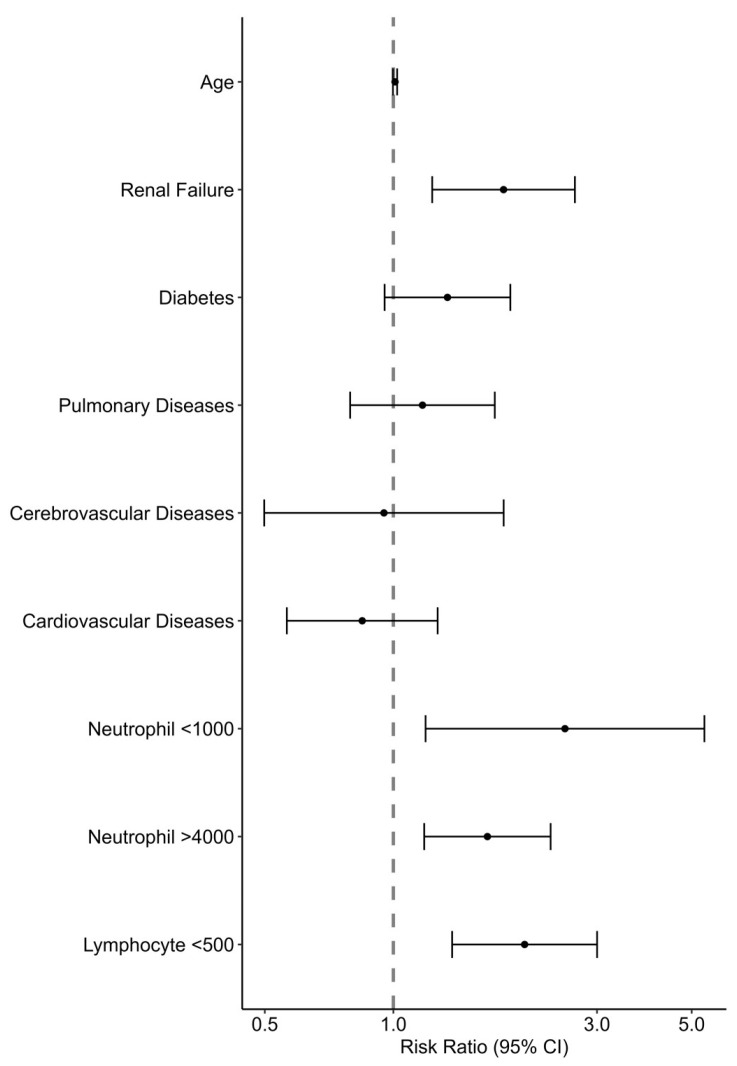
Risk factors associated with the composite poor outcome of in-hospital mortality, mechanical ventilation, and ICU admission among patients hospitalized with RSV: Poisson regression analysis.

**Figure 4:**
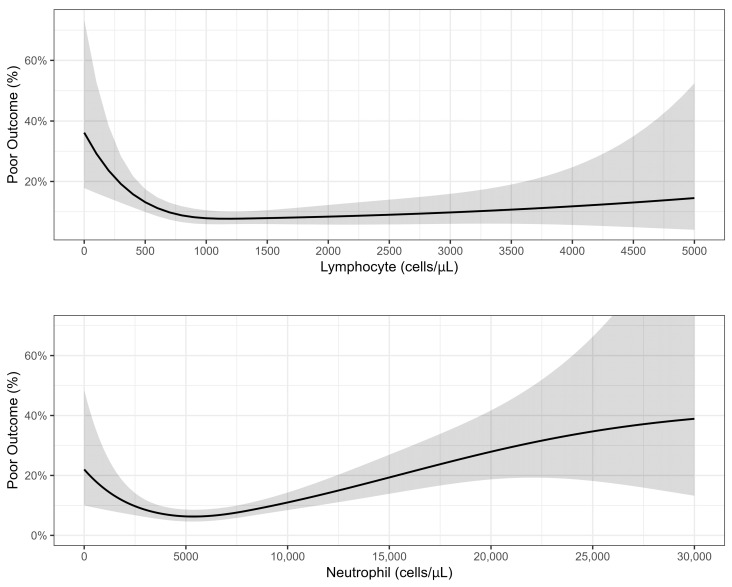
Association between lymphocyte and neutrophil counts and poor outcome: results of quasi-Poisson regression.

On a multivariable analysis (see Figure 3, Supplementary Table S6), significant factors that predicted a poor prognosis were CRF, neutropenia (<1000/μL), neutrophilia (>4000/μL), and lymphopenia (<500/μL). Age and other comorbidities were not found to be predictive of outcomes. The association of a poor outcome with WBC and lymphocyte counts on a continuous scale with the spline set to three degrees of freedom is presented in Figure 4. A lymphocyte count between 0 and 1200 cells/μL was inversely associated with a poor prognosis, while the association with the neutrophil count had a U shape, with the lowest risk at around 5400 cells/μL. 

## 4. Discussion

In this two-center study across seven years, we analyzed data on hospitalized adult patients with RSV infections, calculated population-level incidence rates, assessed risk factors for hospitalization, compared them to those for FLU-associated hospitalization, and assessed parameters predictive of poor outcomes of RSV hospitalization.

The incidence rates for hospitalization with RSV during the study period were overall ~4-fold lower than for FLU (28.2 vs. 101.6/100,000 population). This ratio was similar also for the incidence of the poor composite outcome (2.8 vs. 12.1/100,000). These rates were considerably more prominent in older individuals. For FLU, a significant increase began in middle-aged individuals 55–64 years old, whereas for RSV, an increase was noted after the age of 65 (63.9/100,000) and more so after 75 (199/100,000). Notably, RSV-hospitalized patients were about five years older than FLU patients (see Table S4). This difference might reflect the higher number of pregnant women hospitalized with FLU. Unlike FLU, RSV is an uncommon cause of hospitalization among pregnant women; other studies from developed countries noted similar or higher incidence rates [7,8,9,10,11]. These considerable numbers translate into a nationwide estimate of more than 2775 hospitalizations annually for the population of Israel (9.8 million). The burden on health services, especially hospital occupancy from RSV, is much more significant, considering that RSV hospitalizations are condensed within only ~3–5 months. 

By comparing patients hospitalized with RSV or FLU to the general population, we show that similar factors are associated with hospitalization risk, even after adjusting for age. The highest risk was shown for chronic pulmonary, cardiovascular, and renal diseases, diabetes, hypertension, and lymphoma, with risk ratios of ~3–4 compared to age-adjusted individuals without these comorbidities. A moderately increased risk was found for rheumatologic and liver diseases and solid cancer, with the latter two not reaching statistical significance for RSV patients, most likely due to a smaller number than FLU. Notably, risk ratios were higher for RSV than for FLU for most comorbidities. This finding was also previously reported [12], possibly emphasizing that RSV hospitalization is uncommon in patients with no comorbidities, unlike FLU. 

RSV hospitalization is well-known to be correlated with cardiovascular diseases, both due to the increased risk of symptomatic and severe RSV infection and to an increased cardiovascular events rate following RSV infections, including exacerbation of heart failure, coronary events, and arrhythmias [13,14]. Previous studies showed similar comorbidity risk associations, including chronic cardiovascular, pulmonary, renal, and liver diseases, and diabetes [1,11,15,16,17,18,19,20]. 

Among 942 hospitalized RSV patients, 131 (14%) had a poor outcome, including 37 ICU admissions, 36 patients who were mechanically ventilated, and 59 patients who died during their stay. These patients also had a prolonged average stay of 14.9 days and a rehospitalization rate of 24% among those who survived. We have chosen a composite poor outcome to better reflect the population of hospitalized RSV patients, which also includes a significant proportion of elderly and frail individuals who might be managed with a limited-care strategy of do not intubate/do not resuscitate; thus, in-hospital deaths might not be preceded by mechanical ventilation and ICU care. The same composite outcome was reported for 18.5% of 1634 RSV patients hospitalized in the United States, albeit with a higher rate of ICU admissions and a lower rate of death, possibly reflecting a lower tendency to limit advanced medical care [1]. In-hospital mortality was previously reported as 4.7–7.2% [1,21,22].

Estimating the risk of poor outcomes at the presentation of an RSV patient to the hospital will have significant implications, including decisions on admission and the level of monitoring and care. We used a multivariable model with the demographic data and the patient’s primary clinical and laboratory data on the first day of admission. Surprisingly, age and comorbidities other than renal failure were not predictive risks of poor outcomes in our study, while abnormal hematological indices, including neutrophilia, neutropenia, and lymphopenia, were significantly associated with an increased risk, with RRs of 2.53, 1.66, and 2.03, respectively. Celante et al. reported a correlation between extreme age (≥85) and neutropenia with mortality [21]. Tseng et al. demonstrated a correlation between extreme age, lymphoma, tachypnea ≥ 26/min, decreased consciousness, acute renal failure, and 60-day mortality [22]. We suggest that although many comorbidities increase the risk of RSV hospitalization, the admissions outcomes are more related to the severity of the clinical presentation, including laboratory markers. Lymphopenia is a well-known marker of severity in some viral infections, including FLU and COVID-19 [23]. Neutropenia is probably a marker of recent chemotherapeutic treatment, which could impair immune responses, and neutrophilia might signal a secondary bacterial infection or an exaggerated inflammatory host response.

Bacterial co-infections and super-infections are not uncommon in hospitalized RSV patients. Studies that evaluated co-infections as positive microbiological results of blood and lower respiratory tract specimens in patients with RSV and pneumonia showed various rates between 9.3 and 29% [21,24,25], and one study that separated co-infections (up to day 2 of hospitalization) and super-infections (day ≥ 3), reported rates of 17.1% and 10.3%, respectively [26]. Although we did not directly evaluate bacterial infections, the higher CRP and neutrophil counts in patients with poor prognoses might signify bacterial infections and their impact on the prognosis. In our study, antimicrobial treatment was prescribed as an empiric treatment to 45% of all RSV patients and continued after RSV-PCR results in 28%. This prescribing rate is significantly lower than in previous studies that reported an antibiotic use of 76–94% [21,27,28]. The antimicrobial treatment rate was 57.5% and 53.5% before and after RSV-PCR, respectively, for the patients with poor outcomes, reflecting their worse clinical presentation and possibly higher bacterial co-infections and super-infections. 

The main strength of our study is the completeness of data on RSV hospitalization. As the participating hospitals each serve as the sole hospital in their region, and taking into account the robust RSV-PCR screening strategy employed, we can safely assume that all severe RSV infections leading to hospitalization were captured. We, therefore, can present a valid estimation of the incidence rate. Additionally, using data from almost 1000 hospitalized patients with RSV and comparing them to a population of ~400,000 with detailed electronic health data allowed for a robust calculation of hospitalization risk factors.

Nevertheless, some limitations should be noted. First, as RSV testing was not performed in the community, we cannot estimate the true incidence of RSV in the community, and these data only describe severe cases. Relying only on hospital data for RSV without community-level testing limits our understanding, which may overinflate the perceived risk of RSV in the general population, but also underestimate the overall burden of RSV on medical services. Nonetheless, hospitalization is a significant burden in older adults, representing 14.5–24.5% of community-acquired infections [10,29], and it contributes significantly to healthcare costs [30]. Additional burdens not included in this study include community healthcare visits, emergency department visits without hospitalization, antimicrobial treatments, and late complications of RSV infections such as cardiovascular events. Second, we could only include some comorbidities that could be ascertained with high validity from patients’ health records; therefore, the absence of other comorbidities might have biased our multivariable analyses. The retrospective nature of this study may introduce biases due to incomplete or inaccurate medical records and the inability to control all confounding factors. 

Moving forward, it is essential to prioritize comprehensive surveillance in both hospital and community settings to gain a deeper understanding of the epidemiology and risk factors associated with RSV across varying levels of disease severity. With the introduction of RSV vaccines, it is important to analyze their effects on hospitalization rates and mortality.

## 5. Conclusions

RSV infections are a significant cause of hospitalization in adults, mainly elderly individuals with common comorbidities. Characterizing risk factors for hospitalization can assist in prioritizing high-risk populations for RSV vaccination. Within hospitalized patients with RSV, simple laboratory markers can predict worse outcomes and direct resources such as ICU admission.

## Figures and Tables

**Table 1:** Demographic and clinical characteristics of hospitalized patients with RSV who had a good or poor outcome.

Parameter		All Patients	Good Outcome	Poor Outcome	*p* Value
n		942	811 (86%)	131 (14%)	
Age, median (IQR)		72.9 (62.5–81.8)	72.8 (61.6–81.8)	73.9 (65.9–81.5)	0.237
Age group, n (%)	18–34	44 (4.7%)	43 (5.3%)	1 (0.8%)	0.066
	35–49	66 (7.0%)	60 (7.4%)	6 (4.6%)	
	50–64	161 (17.1%)	139 (17.1%)	22 (16.8%)	
	≥65	671 (71.2%)	569 (70.2%)	102 (77.9%)	
Female sex, n (%)		546 (58.0%)	477 (58.8%)	69 (52.7%)	0.22
Hospital, n (%)	SUMC	780 (82.8%)	678 (83.6%)	102 (77.9%)	0.136
	AAUH	162 (17.2%)	133 (16.4%)	29 (22.1%)	
Comorbidities	Cardiovascular disease, n (%)	201 (21.3%)	170 (21.0%)	31 (23.7%)	0.558
	Cerebrovascular accident, n (%)	57 (6.1%)	48 (5.9%)	9 (6.9%)	0.821
	Pulmonary disease, n (%)	178 (18.9%)	148 (18.2%)	30 (22.9%)	0.254
	Diabetes mellitus, n (%)	327 (34.7%)	269 (33.2%)	58 (44.3%)	0.017
	Chronic renal failure, n (%)	158 (16.8%)	122 (15.0%)	36 (27.5%)	0.001
Clinical presentation at ED	Fever, n (%)	229 (26.9)	206 (27.7)	23 (21.5)	0.214
	Hypoxemia, n (%)	318 (37.7)	268 (36.5)	50 (45.5)	0.089
WBC, cells/μL, median (IQR)		8.6 (6.5–11.6)	8.4 (6.5–11.3)	9.9 (6.3–14.8)	0.016
WBC grouping	Normal, n (%)	566 (60.1%)	504 (62.1%)	62 (47.3%)	0.004
	Low, n (%)	88 (9.3%)	74 (9.1%)	14 (10.7%)	
	High, n (%)	288 (30.6%)	233 (28.7%)	55 (42.0%)	
Neutrophils, cells/μL, median (IQR)		6.6 (4.5–9.6)	6.4 (4.5–9.3)	8.0 (4.6–12.7)	0.002
Neutrophil grouping	Normal, n (%)	499 (53.0%)	447 (55.1%)	52 (39.7%)	0.002
	Low, n (%)	28 (3.0%)	21 (2.6%)	7 (5.3%)	
	High, n (%)	415 (44.1%)	343 (42.3%)	72 (55.0%)	
Lymphocytes, cells/μL, median (IQR)		1.1 (0.7–1.6)	1.1 (0.7–1.6)	0.9 (0.50–1.4)	0.002
Lymphocyte grouping	Normal, n (%)	500 (53.1%)	444 (54.7%)	56 (42.7%)	0.026
	Low, n (%)	435 (46.2%)	362 (44.6%)	73 (55.7%)	
	High, n (%)	7 (0.7%)	5 (0.6%)	2 (1.5%)	
C-reactive protein grouping	<50 mg/L	387 (51.5%)	338 (53.1%)	49 (42.6%)	0.017
	50–100 mg/dL	170 (22.6%)	146 (22.9%)	24 (20.9%)	
	>100 mg/dL	195 (25.9%)	153 (24.0%)	42 (36.5%)	
Antimicrobial treatment, n (%)	Before PCR results	418 (44.8%)	345 (42.8%)	73 (57.5%)	0.003
	After PCR results	260 (27.8%)	192 (23.8%)	68 (53.5%)	<0.001
ICU admission, n (%)		37 (3.9%)	0 (0.0%)	37 (28.2%)	
Mechanical ventilation, n (%)		36 (3.8%)	0 (0%)	36 (27/5%)	
In-hospital death, n (%)		59 (15.1%)	0 (0.0%)	59 (45.0%)	
Length of stay, days, mean (SD)		5.9 (10.8)	4.5 (8.4)	14.9 (17.5)	<0.001
Length of stay <3 days, n (%)		573 (60.8%)	544 (67.1%)	29 (22.1%)	<0.001
Rehospitalization within 30 days, n (%)		84 (8.9%)	67 (8.3%)	17 (13.0%)	0.111

Abbreviations: IQR, interquartile range; SUMC, Soroka University Medical Center; AAUH, Assuta Ashdod University Hospital; ED, emergency department; WBC, white blood cells; ICU, intensive care unit; SD, standard deviation.

## Data Availability

De-identified data are available upon request from the corresponding author.

## Supplementary Materials

The following supporting information can be downloaded at: https://www.mdpi.com/article/10.3390/pathogens13090750/s1: Table S1: ICD-9-CM and ICD-10 Coding Algorithms for Elixhauser Comorbidities; Table S2: Mean incidence rates for hospitalization of adults with Respiratory syncytial virus (RSV) and influenza (FLU), 2016–2022; Table S3: Characteristics of Clalit Health Services’ Insured Population in the Southern District and hospitalized patients with respiratory syncytial virus infection and influenza at Soroka University Medical Center, 2017–2022; Table S4: Comparative demographic and clinical profile of influenza and respiratory syncytial virus cases, SUMC and AAUH, 2017–2022; Table S5: Risk Factors Associated with Hospitalization due to Influenza and Respiratory Syncytial Virus, Adjusted for Age; Table S6: Multivariable analysis evaluating risk factors for poor outcomes among hospitalized patients with RSV.
