# Supplementary material for: Adult Respiratory Syncytial Virus Infection: Defining Incidence, Risk Factors for Hospitalization, and Poor Outcomes, a Regional Cohort Study, 2016–2022
Source: Pathogens. 2024 Aug 31;13(9):750. doi: 10.3390/pathogens13090750 (PMC11434971; doi:10.3390/pathogens13090750)
Supplement: Supplementary file 1 [file pathogens-13-00750-s001.zip › pathogens-3172688-supplementary.pdf]

Adult Respiratory Syncytial Virus Infection: Defining Incidence, Risk Factors for Hospitalization, and Poor Outcomes, a Regional Cohort Study, 2016–2022

Supplementary material

Table of contents

Page number	Table
2	Table S1: ICD-9-CM and ICD-10 Coding Algorithms for Elixhauser Comorbidities
6	Table S2: Mean incidence rates for hospitalization of adults with Respiratory syncytial virus (RSV) and influenza (FLU), 2016-2022
7	Table S3: Characteristics of Clalit Health Services' Insured Population in the Southern District and hospitalized patients with respiratory syncytial virus infection and influenza at Soroka University Medical Center, 2017-2022
8	Table S4: Comparative demographic and clinical profile of influenza and respiratory syncytial virus cases, SUMC and AAUH, 2017-2022
9	Table S5. Risk Factors Associated with Hospitalization due to Influenza and Respiratory Syncytial Virus, Adjusted for Age
10	Table S6: Multivariable analysis evaluating risk factors for poor outcomes among hospitalized patients with RSV

Table S1. ICD-9-CM and ICD-10 Coding Algorithms for Elixhauser Comorbidities

Comorbidities	Group	Elixhauser's original ICD-9-CM	Elixhauser AHRQ- Web ICD-9-CM	ICD-10	Enhanced ICD-9-CM
Congestive heart failure	Cardiovascular Diseases	398.91, 402.11, 402.91, 404.11, 404.13, 404.91, 404.93, 428.x	398.91, 402.11, 402.01, 404.03, 402.91, 404.91, 404.01, 404.11, 404.13, 404.93, 428.x	I09.9, I11.0, I13.0, I13.2, I25.5, I42.0, 142.5-I42.9, I43.x, I50.x, P29.0	398.91, 402.01, 402.11. 402.91, 404.01, 404.03, 404.11, 404.13, 404.91, 404.93, 425.4-425.9, 428.x
Cardiac arrhythmias		426.10, 426.11, 426.13, 426.2-426.53, 426.6-426.8, 427.0, 427.2, 427.31, 427.60, 427.9, 785.0, V45.0, V53.3		I44.1-I44.3, I45.6, I45.9, I47.x-I49.x, ROO.O, ROO.1, ROO.8, T82.1, Z45.0, Z95.0	426.0, 426.13, 426.7, 426.9, 426.10, 426.12, 427.0-427.4, 427.6-427.9, 785.0, 996.01, 996.04, V45.0, V53.3
Valvular disease		093.2, 394.0-397.1, 424.0-424.91, 746.3-746.6, V42.2, V43.3	093.2, 394.x-397.1, 397.9, 424.x, 746.3-746.6, V42.2, V43.3	A52.0, I05.x-I08.x, I09.1, I09.8, I34.x-I39.x, Q23.O-Q23.3, Z95.2, Z95.4	093.2, 394.x-397.x, 424.x, 746.3-746.6, V42.2, V43.3
Pulmonary circulation Disorders	Pulmonary Diseases	416.x, 417.9	416.x, 417.9	I26.x, I27.x, I28.0, I28.8, I28.9	415.0, 415.1, 416.x, 417.0, 417.8, 417.9
Peripheral vascular disorders		440.x, 441.2, 441.4, 441.7, 441.9, 443.1- 443.9, 447.1, 557.1, 557.9, V43.4	440.x, 441.x, 442.x, 443.1-443.9, 447.1, 557.1, 557.9, V43.4	I70.x, I71.x, I73.1, I73.8, I73.9, I77.1, I79.0, I79.2, K55.1, K55.8, K55.9, Z95.8, Z95.9	093.0, 437.3, 440.x, 441.x, 443.1-443.9, 447.1, 557.1 557.9, V43.4
Hypertension, uncomplicated	Hypertension	401.1, 401.9	401.1, 401.9, 642.0	I10.x	401.x
Hypertension, complicated		402.10, 402.90, 404.10, 404.90, 405.1, 405.9	401.0, 402.x-405.x, 642.1, 642.2, 642.7, 642.9	I11.x-I13.x, I15.x	402.x-405.x
Paralysis		342.0. 342.1, 342.9-344.x	342.x-344.x, 438.2-438.5	G04.1, G11.4, G80.1, G80.2, G81.x, G82.x, G83.0-G83.4, G83.9	334.1, 342.x, 343.x, 344.0-344.6, 344.9
Other neurological disorders		331.9, 332.0, 333.4, 333.5, 334.x, 335.x, 340.x, 341.1-341.9, 345.0, 345.1, 345.4, 345.5, 345.8, 345.9, 348.1, 348.3, 780.3, 784.3	330.x-331.x. 332.0, 333.4, 333.5, 334.x, 335.x, 340, 341.1-341.9, 345.x, 347.x, 780.3, 784.3	G10.x-G 13.x, G20.x-G22.x, G25.4, G25.5, G31.2, G31.8, G31.9, G32.x, G35.x-G37.x, G40.x, G41.x, G93.1, G93.4, R47.0, R56.x	331.9, 332.0, 332.1, 333.4, 333.5, 333.92, 334.x-335.x, 336.2, 340.x, 341.x, 345.x, 348.1, 348.3, 780.3, 784.3
Chronic pulmonary disease	Pulmonary Diseases	490-492.8, 493.00-493.91, 494.x-505.x, 506.4	490x-492.x, 493.x, 494x-505.x, 506.4	I27.8, 127.9, J40.x-J47.x, J60.x-J67.x, J68.4, J70.1, J70.3	416.8, 416.9, 490.x-505.x, 506.4, 508.1, 508.8
Diabetes, uncomplicated	Diabetes	250.0-250.3	250.0-250.3, 648.0	E10.0, E10.1, E10.9, E11.0, E11.1, E11.9, E12.0, E12.1, E12.9, E13.0, E13.1, E13.9, E14.0, E14.1, E14.9	250.0-250.3
Diabetes, complicated		250.4-250.7, 250.9	250.4-250.9, 775.1	E10.2-E10.8,	250.4-250.9

				E11.2-E11.8, E12.2-E12.8, E13.2-E13.8, E14.2-E14.8	
Hypothyroidism		243-244.2, 244.8, 244.9	243-244.2, 244.8, 244.9	E00.x-E03.x, E89.0	240.9, 243.x, 244.x, 246.1, 246.8
Renal failure		403.11, 403.91, 404.12, 404.92, 585.x, 586.x, V42.0, V45.1, V56.0, V56.8	403.01, 403.91, 403.11, 404.02, 404.12, 404.03, 404.13, 404.93, 404.92, 585.x, V42.0, 586 .x, V45.1, V56.x	I12.0, I13.1, N18.x, NI9.x, N25.0, Z49.0-Z49.2, Z94.0, Z[1]99.2	403.01, 403.11, 403.91, 404.02, 404.03, 404.12, 404.13, 404.92, 404.93, 585.x, 586.x, 588.0, V42.0, V45.1, V56.x
Liver disease		070.32, 070.33, 070.54, 456.0, 456.1, 456.2, 571.0, 571.2-571.9, 572.3, 572.8, V42.7	070.22, 070.23, 070.32, 070.33, 070.44, 070.54, 456.0, 456.1, 456.20, 571.0, 571.2-571.9, 572.3, 572.8, V42.7	B18.x, I85.x, I86.4, I98.2, K70.x, K71.1, K71.3-K71.5, K71.7, K72.x-K74.x, K76.0, K76.2-K76.9. Z94.4	070.22, 070.23, 070.32, 070.33, 070.44, 070.54, 070.6, 070.9, 456.0-456.2, 570.x, 571.x, 572.2-572.8, 573.3, 573.4, 573.8,573.9, V42.7

**Continued** 4					
**Comorbidities**	**Group**	**Elixhauser's original ICD-9-CM**	**Elixhauser AHRQ-Web ICD-9-CM**	**ICD-10**	**Enhanced ICD-9-CM**
Peptic ulcer disease excluding bleeding		531.70, 531.90, 532.70, 532.90, 533.70, 533.90, 534.70, 534.90, V12.71	531.41, 531.51, 531.61, 531.7, 531.91, 532.41, 532.51, 532.61, 532.7, 532.91, 533.41, 533.51, 533.61, 533.7, 533.91, 534.41, 534.51, 534.61, 534.7, 534.91	K25.7, K25.9, K26.7, K26.9, K27.7, K27.9, K28.7, K28.9	531.7, 531.9, 532.7, 532.9, 533.7, 533.9, 534.7, 534.9
AIDS/H1V		042.x-044.x	042.x-044.x	B20.x-B22.x, B24.x	042.x-044.x
Lymphoma		200.x-202.3x, 202.5-203.0, 203.8, 238.6, 273.3, V10.71, V10.72, V10.79	200.x-202.3, 202.5-203.0, 203.8, 238.6, 273.3	C81.x-C85.x, C88.x, C96.x, C90.0, C90.2	200.x-202.x, 203.0, 238.6
Metastatic cancer	Solid Tumor	196.x-199.x	196.x-199.x	C77.x-C80.x	196.x-199.x
Solid tumor without metastasis		140.x-172.x, 174.x, 175.x, 179.x-195.x, V10.x	140.x-172.x, 174.x, 175.x, 179.x-195.x	C00.x-C26.x, C30.x-C34.x, C37.x-C41.x, C43.x, C45.x-C58.x, C60.x-C76.x, C97.x	140.x-172.x, 174.x-195.x
Rheumatoid arthritis/ collagen vascular diseases		701.0, 710.x, 714.x, 720.x, 725.x	701.0, 710.x, 714.x, 720.x, 725.x	L94.0, L94.1, L94.3, M05.x, M06.x, M08.x, M12.0, M12.3, M30.x, M31.0-M31.3, M32.x-M35.x, M45.x, M46.1, M46.8, M46.9	446.x, 701.0, 710.0-710.4, 710.8, 710.9, 711.2, 714.x, 719.3, 720.x, 725.x, 728.5, 728.89, 729.30
Coagulopathy		286.x, 287.1, 287.3-287.5	286.x, 287.1, 287.3-287.5	D65-D68.x, D69.1, D69.3-D69.6	286.x, 287.1, 287.3-287.5
Obesity		278.0	278.0	E66.x	278.0
Weight loss		260.x-263.x	260.x-263.x, 783.2	E40.x-E46.x, R63.4, R64	260.x-263.x, 783.2, 799.4
Fluid and electrolyte disorders		276.x	276.x	E22.2, E86.x, E87.x	253.6, 276.x
Blood loss anemia		280.0	280.0, 648.2	D50.0	280.0
Deficiency anemia		280.1-281.9, 285.9	280.1-281.9, 285.2, 285.9	D50.8, D50.9, D51.x-D53.x	280.1-280.9, 281.x
Alcohol abuse		291.1, 291.2, 291.5-291.9, 303.9, 305.0, V113	291.0-291.3, 291.5, 291.8, 291.9, 303.x, 305.0	F10, E52, G62.1, I42.6, K29.2, K70.0, K70.3, K70.9, T51.x, Z50.2, Z71.4, Z72.1	265.2, 291.1-291.3, 291.5-291.9, 303.0, 303.9, 305.0, 357.5, 425.5, 535.3, 571.0-571.3, 980.x, V11.3
Drug abuse		292.0, 292.82-292.89, 292.9, 304.0, 305.2, 305.9	292.0, 292.82-292.89, 292.9, 304.x, 305.2-305.9, 648.3	F11.x-F16.x, F18.x, F19.x, Z71.5. Z72.2	292.x, 304.x, 305.2-305.9, V65.42

Psychoses		295.x-298.x, 299.1	295.x-298.x, 299.1	F20.x, F22.x-F25.x, F28.x, F29.x, F30.2, F31.2, F31.5	293.8, 295.x, 296.04, 296.14, 296.44, 296.54, 297.x, 298.x
Depression		300.4, 301.12, 309.0, 309.1, 311	300.4, 301.12, 309.0, 309.1, 311	F20.4, F31.3-F31.5, F32.x, F33.x, F34.1, F41.2, F43.2	296.2, 296.3, 296.5, 300.4, 309.x, 311

Source:

Quan H, Sundararajan V, Halfon P, et al. Coding algorithms for defining Comorbidities in ICD-9-CM and ICD-10 administrative data. *Med Care.* 2005 Nov; 43(11): 1130-9.

Table S2: Mean incidence rates for hospitalization of adults with Respiratory syncytial virus (RSV) and influenza (FLU), 2016-2022

Age group, years	RSV incidence/100,000 population, median (IQR)	FLU incidence/100,000 population, median (IQR)
20-24	2.5 (1.3-2.7)	40.0 (22-52.8)
25-29	2.9 (1.4-2.9)	36.6 (23.8-49.5)
30-34	3.3 (3.1-4.8)	35.7 (34.7-46.8)
35-44	5.3 (1.8-5.7)	23.3 (22.4-45.5)
45-54	12.5 (8.3-13.4)	62 (33.2-64.7)
55-64	19 (14.3-29.7)	117.2 (74.6-118.7)
65-74	63.9 (36.3-78.3)	217.1 (148.3-236.5)
≥75	199 (79.2-202.5)	411.8 (387-565.4)

Abbreviations: RSV, respiratory syncytial virus; FLU, influenza; IQR, interquartile range

Table S3: Characteristics of Clalit Health Services' Insured Population in the Southern District and hospitalized patients with respiratory syncytial virus infection and influenza at Soroka University Medical Center, 2017-2021

		CHS insured population	Hospitalized patients with influenza	Hospitalized patients with RSV
N		1,858,742	1,462	411
Male sex, N (%)		885,058 (47.6%)	695 (47.5%)	168 (40.9%)
Jewish ethnicity, n (%)		1,365,545 (73.5%)	1,012 (69.2%)	311 (75.7%)
Season, n (%)	2017/18	353,398 (19%)	533 (36.5%)	118 (28.7%)
	2018/19	362,058 (19.5%)	364 (24.9%)	111 (27%)
	2019/20	370,644 (19.9%)	313 (21.4%)	106 (25.8%)
	2020/21	380,702 (20.5%)	0 (0%)	18 (4.4%)
	2021/22	391,940 (21.1%)	252 (17.2%)	58 (14.1%)
Comorbidity, n (%)	any	759,091 (40.8%)	1,224 (83.7%)	377 (91.7%)
	Cardiovascular disease	203,094 (10.9%)	638 (43.6%)	222 (54%)
	Pulmonary disease	258,772 (13.9%)	660 (45.1%)	227 (55.2%)
	Hypertension	386,191 (20.8%)	880 (60.2%)	309 (75.2%)
	Diabetes mellitus	164,377 (8.8%)	510 (34.9%)	173 (42.1%)
	Renal failure	80,398 (4.3%)	350 (23.9%)	135 (32.8%)
	Liver disease	142,203 (7.7%)	232 (15.9%)	58 (14.1%)
	Lymphoma	14,928 (0.8%)	49 (3.4%)	15 (3.6%)
	Solid tumor	89,279 (4.8%)	200 (13.7%)	62 (15.1%)
	Rheumatological disease	76,555 (4.1%)	135 (9.2%)	45 (10.9%)

Table S4: Comparative demographic and clinical profile of influenza and respiratory syncytial virus cases, SUMC and AAUH, 2017-2022

Parameter		FLU	RSV	P value
N		1821	520	
Age, years, median (IQR)		68.4 (52.3-79.4)	73.6 (62.6-81.8)	<0.001
Male sex, n (%)		861 (47.3%)	207 (39.8%)	0.003
Jewish, n (%)		1226 (67.3%)	389 (74.8%)	0.001
Socioeconomic score, n (%)	Very high	49 (2.7%)	15 (2.9%)	0.049
	High	224 (12.3%)	69 (13.3%)	
	Medium	410 (22.5%)	132 (25.4%)	
	Low	459 (25.2%)	151 (29%)	
	Very low	367 (20.2%)	82 (15.8%)	
	No data	312 (17.1%)	71 (13.7%)	
Comorbidities, n (%)	Any	1511 (83%)	475 (91.3%)	<0.001
	Cariovascular disease	767 (42.1%)	284 (54.6%)	<0.001
	Pulmonary disease	824 (45.2%)	284 (54.6%)	<0.001
	Hypertension	1086 (59.6%)	390 (75%)	<0.001
	Diabetes mellitus	629 (34.5%)	227 (43.7%)	<0.001
	Chronic renal failure	423 (23.2%)	167 (32.1%)	<0.001
	Liver disease	296 (16.3%)	81 (15.6%)	0.762
	Lymphoma	58 (3.2%)	23 (4.4%)	0.220
	Rheumatological disease	174 (9.6%)	61 (11.7%)	0.170

Abbreviations: SUMC, Soroka University Medical Center; AAUH, Assuta Ashdod University Hospital

Table S5. Risk Factors Associated with Hospitalization due to Influenza and Respiratory Syncytial Virus, Adjusted for Age

	RSV		Influenza	
Comorbidity	Relative risk (95% CI)	P	Relative risk (95% CI)	P
Cardiovascular diseases	3.3 (2.6-4.2)	<0.001	2.7 (2.4-3)	<0.001
Chronic pulmonary diseases	4.2 (3.4-5.2)	<0.001	3.2 (2.9-3.6)	<0.001
Hypertension	2.9 (2.2-3.8)	<0.001	1.8 (1.5-2)	<0.001
Diabetes mellitus	2.4 (1.9-3)	<0.001	2.1 (1.9-2.4)	<0.001
Chronic renal failure	2.9 (2.3-3.7)	<0.001	2.3 (2.-2.6)	<0.001
Lymphoma	2.2 (1.3-3.6)	0.004	2.2 (1.7-3)	<0.001
Rheumatologic diseases	1.2 (0.9-1.7)	0.172	1.2 (1-1.4)	0.082
Solid tumor	1.1 (0.8-1.4)	0.628	1.2 (1-1.36)	0.065
Liver disease	1.1 (0.8-1.4)	0.681	1.3 (1.1-1.5)	<0.001

Table S6: Multivariable analysis evaluating risk factors for poor outcomes among hospitalized patients with RSV

**Parameter**	**Relative risk (95% CI)**	**P**
Age (per year)	1.01 (0.998-1.02)	0.115
Cardiovascular diseases	0.85 (0.56-1.27)	0.419
Pulmonary diseases	1.17 (0.79-1.73)	0.878
Diabetes mellitus	1.34 (0.95-1.88)	0.092
**Renal Failure**	**1.81 (1.23-2.66)**	**0.003**
**Neutrophil count <1000/μL**	**2.53 (1.19-5.35)**	**0.016**
**Neutrophil count >4000/μL**	**1.66 (1.81-2.34)**	**0.004**
**Lymphocyte count<500**	**2.03 (1.37-3.0)**	**<0.001**
